# On the Error Metrics Used for Direction of Arrival Estimation

**DOI:** 10.3390/s25082358

**Published:** 2025-04-08

**Authors:** Mohammad Abdul Hannan, Ottavio Crisafulli, Giuseppe Giammello, Gino Sorbello

**Affiliations:** 1 Department of Electrical, Electronics and Computer Engineering, University of Catania, I-95123 Catania, Italy; giuseppe.giammello@phd.unict.it (G.G.); gino.sorbello@unict.it (G.S.); 2 Consorzio Nazionale Interuniversitario per le Telecomunicazioni, Research Unit University of Catania, I-95123 Catania, Italy; ottavio.crisafulli@cnit.it; 3 Department of Information Engineering, Infrastructure and Sustainable Energy, Mediterranea University of Reggio Calabria, I-89122 Reggio Calabria, Italy

**Keywords:** DoA estimation, 2D DoA, RMSE, MSE, MAE, error metrics

## Abstract

In this article, the error metrics used for evaluating the performance of direction of arrival (DoA) estimation are thoroughly investigated to recommend the most suitable one. This investigation highlights the lack of consensus in the literature regarding the selection and definition of these metrics. We show that this disparity is particularly serious in 2D DoA estimation, an aspect often overlooked by many authors. Notably, certain widely accepted error metrics can yield inaccurate and misleading results. Therefore, this article advocates for the adoption of a specific error metric that ensures accurate and meaningful assessments of 2D DoA estimation. A set of numerical and experimental results is presented to demonstrate the potential of the proposed error metric compared to other well-known metrics. Unlike other metrics, our proposed error definition is frame-independent. Finally, practical use cases are briefly discussed to highlight the pervasive impact of this fundamental definition.

## 1. Introduction

Direction of arrival (DoA) estimation is a long-standing area of research extensively studied in various disciplines and fruitfully applied in many fields of engineering, including in radar systems [1], sonar, navigation, geophysical and seismic sensing, next-generation wireless communications and sensing [2,3], the internet of things (IoTs), vehicular technology [4], and unmanned aerial vehicles (UAVs) [5], to name a few. As a matter of fact, a significant number of publications exist on the state-of-the-art (SoA) literature on DoA estimation. To provide some insights on the number of publications on this topic, the keyword “direction of arrival estimation” was anonymously searched for in Scopus (https://www.scopus.com/ (accessed on 17 June 2024)) and IEEE Explore (https://ieeexplore.ieee.org/Xplore/home.jsp (accessed on 17 June 2024)) at 14:00 (GMT+2). It is worth reporting that there are a total of 8845 and 4375 journals, 8153 and 10,633 conferences, 257 and 237 other publications (i.e., manuscripts outside of journals and conferences) that have been published in Scopus and IEEE Explore, respectively.

**Figure 1:**
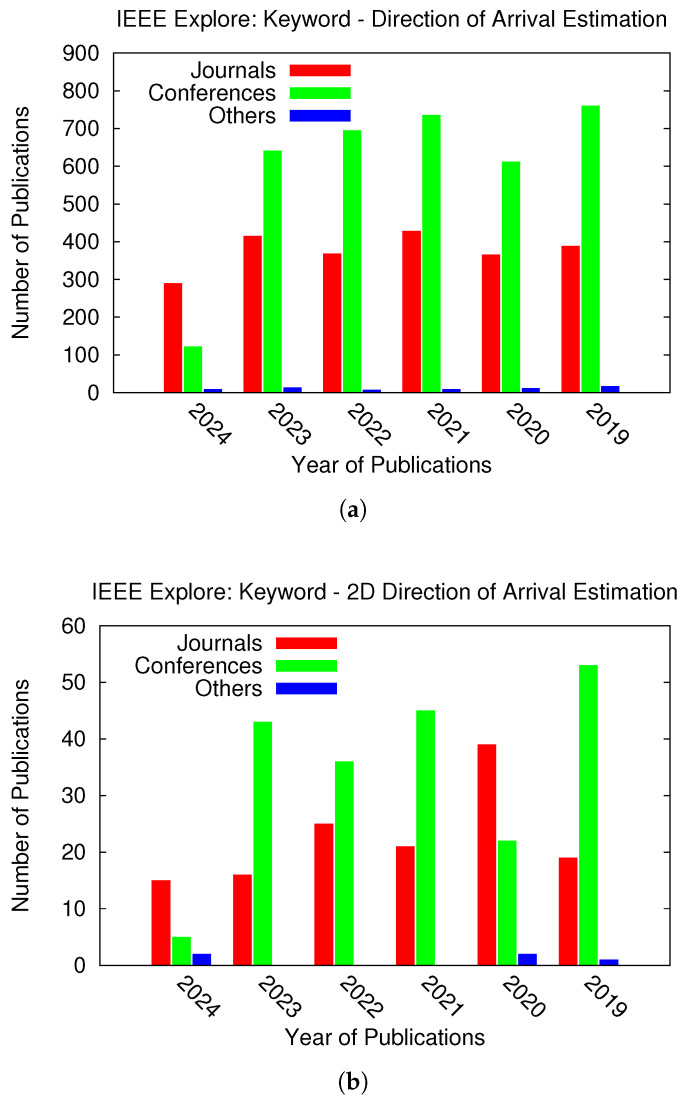
The number of publications in the last five years in IEEE Explore containing the keywords (**a**) “Direction of Arrival Estimation” and (**b**) “2D Direction of Arrival Estimation”.

Although it is a mature topic, it has become a research area of great interest at present due to the recent advancements in (i) signal processing [4] and machine learning techniques [6,7,8]; (ii) electronics and sensing devices [9,10]; and (iii) wireless technologies [2,3] and services [5,11]. The number of manuscripts published on “direction of arrival estimation” (Figure 1a) and “2D direction of arrival estimation” (Figure 1b) in the last six years in IEEE Explore is shown in Figure 1. Evidently, an average of about 400 journals per year have published articles on DoA estimation, while 25 of them have focused on 2D DoA cases.

Therefore, more works on 2D DoA estimation are expected in near future, not only because the number of publications on 2D DoA estimation has increased each year since 2021, but also because of the great demand coming from the recent advances in wireless and communication technologies, including 5G/6G [12].

Throughout the SoA literature on DoA estimation, it is very common to evaluate the performance of these estimations with the Cramér–Rao Bound (CRB) and/or with an error metric. Although they are both related to the performance of the estimators, they serve different purposes in the context of statistical estimation and signal processing. The key differences between them are summarized below:

- The CRB serves as a theoretical benchmark for the variance of unbiased estimators, whereas error metrics are practical tools used to assess the accuracy of any estimator, regardless of whether it is biased or unbiased;
- The CRB focuses on the minimum achievable variance, offering insight into the optimal performance of an estimator. In contrast, error metrics concentrate on the actual performance of the estimator, providing a measure of how well it performs with real data.

It is worth mentioning that the optimal achievable performance of different DoA estimators is well defined by means of the CRB in the literature. However, the actual performance of DoA estimators is evaluated using different error metrics, namely the mean absolute error (MAE) or mean square error (MSE) or root mean square error (RMSE).

**Figure 2:**
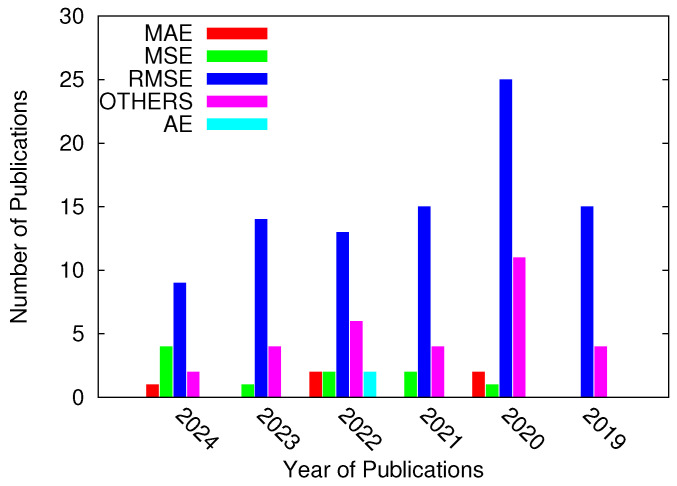
Error metrics used to assess 2D DoA estimation in last six years.

By checking the error metrics used for 2D DoA estimation in the analyzed literature (i.e., journals) from 2019 to 2024 (Figure 1b), the RMSE is the most widely used error metric (i.e., in 91 out of 139 papers), as shown in Figure 2. To provide more detail, we have highlighted the following facts:

- The definition of the error metric used was included in about 22.5% papers. Otherwise, only the names of the error metrics used have been mentioned (e.g., MSE in [13] and RMSE in [14,15,16]).
- Rather than defining a joint error metric, the azimuth and elevation error metrics have been separately defined in about 26% papers, following a pattern akin to that of 1D DoA estimation (e.g., [17,18,19,20]), thus resulting in a “conditional definition”.
- The error metric that combines the azimuth and elevation error has been defined in about 28% papers (e.g., [8,9,10,21,22,23,24,25,26,27]) which is mathematically correct but incorrect in some real scenarios.
- Only two (i.e., 1.5%) papers reported an error metric that combined both the θ and ϕ angular directions by evaluating the angle between the actual direction of arrival $\underline{u\;}\;$ and the estimated one $\hat{\underline{u\;}\;}$. As a matter of fact, this error metric is meaningful from both a mathematical and physical point of view.
- In the roughly 22% papers remaining, the error metrics classified as “OTHERS” in Figure 2 were introduced. This category encompasses various definitions, including the cumulative distributed function (CDF) of the localization error [11] and other less-known/less widespread definitions (e.g., [28]), which are very problem-specific and can not be easily used as a figure of merit for performance comparisons.

As can be inferred, there is no unique or largely adopted error metric for 2D DoA estimation, hence it seems useful to base a study on the identification of the most effective one. To this end, this article aims to analyze in depth popularly used error metrics to recommend a meaningful and versatile error metric for 2D DoA estimation. In summary, the key contributions of this paper are as follows:

- A comprehensive literature review on error metrics used for 1D and 2D DoA estimation.
- A statistical analysis of the research trends in DoA estimation over the past six years, highlighting its growing significance.
- A statistical review of error metric choices in recent studies, revealing the lack of consensus among researchers.
- A mathematical derivation of commonly used error metrics, progressing from simple error estimation to DoA estimation, considering the number of signals, Monte Carlo simulations (trials), and both 1D and 2D cases.
- A numerical analysis of various error metrics applied to test cases, demonstrating inconsistencies between the estimated and observable errors.
- The proposal of a new error metric for 2D DoA estimation that accounts for the problem’s inherent 3D nature, ensuring consistency between the estimated and physical errors.
- A validation of the proposed error metric using both numerical and experimental data, and a comparison of the new metric with existing metrics.
- A discussion on current and potential use cases, emphasizing the importance of properly defining error metrics in DoA estimation.

The rest of the paper is organized as follows: In Section 2, the most commonly-used error metrics for 1D DoA are listed and defined. In Section 3, an in-depth analysis of each metric used for 2D DoA estimation is provided. In Section 4, the sensitivity of the error metrics used for 2D DoA estimation is analyzed using experimental data. Section 5 includes some use cases of the error metrics. Finally, some conclusions are drawn in Section 6.

## 2. Error Metrics for 1D DoA Estimation

Let us start with the following definitions:

(1) $$\begin{array}{c} \Psi_{E}=\hat{\psi }-\psi \end{array}$$

(2) $$\begin{array}{c} \Psi_{\mathrm{SE}}=\Psi_{E}^{2} \end{array}$$

(3) $$\begin{array}{c} \Psi_{\mathrm{RSE}}=\sqrt{\Psi_{\mathrm{SE}}}=\left|\Psi_{E}\right| \end{array}$$

where Ψ is called the estimation error between the true direction of arrival (DoA), ψ, and the estimated one, $\hat{\psi }$. The subscripts of Ψ may indicate the name of an error metric, depending on its mathematical formulation (i.e., $\Psi_{E}$, $\Psi_{\mathrm{SE}}$, and $\Psi_{\mathrm{RSE}}$ may refer to error, square error, and root square error, respectively). The error values (1) and (3) are expressed with the same units as ψ—in either degrees or radians. Moreover, the following observations can be drawn: (i) $\Psi_{E}$ (1) is the straightforward way of defining the error; (ii) the $\Psi_{\mathrm{SE}}$ in (2) seems to be an unconventional definition for a metric since it is not suitable for quantitative error assessments; and (iii) $\Psi_{\mathrm{RSE}}$ (3) may not be the most intuitive error metric, but it is widely adopted since it transforms the $\Psi_{\mathrm{SE}}$ into a quantity with appropriate measurement unit, making its value physically understandable.

In order to gain more insights into the error figures in (1)–(3), a numerical analysis of three simple test cases (TCs) has been performed and the results are summarized in Table 1. As expected, $\Psi_{E}$ and $\Psi_{\mathrm{RSE}}$ correctly estimate the error value in all cases, whereas $\Psi_{\mathrm{SE}}$ is not proportional to the error. Moreover, only $\Psi_{E}$ gives the sign of the error; that is, the direction of the deviation from the actual value. Therefore, the following questions arise:

- Why is $\Psi_{E}$ (1) not widely used?
- Why is $\Psi_{\mathrm{SE}}$ (2) even considered an error metric?
- Why is $\Psi_{\mathrm{RSE}}$ (3) widely used?

To answer these questions, let us consider the case of *M* directions of arrival (i.e., *M* sources, thus a multiple-value estimation problem) and let us extend the definitions of $\Psi_{E}$, $\Psi_{\mathrm{SE}}$, and $\Psi_{\mathrm{RSE}}$ as follows:

(4) $$\begin{array}{c} \Psi_{\mathrm{AE}}=\frac{1}{M}\sum_{m=1}^{M}\left({\hat{\psi }}_{m}-\psi_{m}\right) \end{array}$$

(5) $$\begin{array}{c} \Psi_{\mathrm{ASE}}=\frac{1}{M}\sum_{m=1}^{M}{\left({\hat{\psi }}_{m}-\psi_{m}\right)}^{2} \end{array}$$

(6) $$\begin{array}{ccc} \Psi_{\mathrm{RMSE},a} & = & \frac{1}{M}\sum_{m=1}^{M}\sqrt{{\left({\hat{\psi }}_{m}-\psi_{m}\right)}^{2}}\\ \; & = & \frac{1}{M}\sum_{m=1}^{M}\left|\left({\hat{\psi }}_{m}-\psi_{m}\right)\right|\\ \Psi_{\mathrm{RMSE},b} & = & \sqrt{\frac{1}{M}\sum_{m=1}^{M}{\left({\hat{\psi }}_{m}-\psi_{m}\right)}^{2}}. \end{array}$$

Thus, $\Psi_{\mathrm{AE}}$ (4) is an average error (AE) metric, $\Psi_{\mathrm{ASE}}$ (5) is an average square error (ASE) metric, and $\Psi_{\mathrm{RMSE},a}$ and $\Psi_{\mathrm{RMSE},b}$ of (6) are root mean square error (RMSE) metrics.

Then, let us analyze the results in Table 2 of the numerical analysis when M=3. It turns out that only $\Psi_{\mathrm{RMSE},a}$ in (6), that is, the extended version of $\Psi_{\mathrm{RSE}}$ to multiple-value estimation problems, provides the correct estimation of the error in all cases tested, while $\Psi_{\mathrm{AE}}$ (i.e., the extended version of $\Psi_{E}$) fails to for Case 3. As a result, $\Psi_{E}$ (1) and $\Psi_{\mathrm{AE}}$ (4) are not widely used. Regarding $\Psi_{\mathrm{SE}}$ (2) and $\Psi_{\mathrm{ASE}}$ (5), they work more as figures of merit and penalize larger errors more than a smaller ones (i.e., they may be suitable for a specific optimization problem but not appropriate for defining a general quantitative error metric). On the other hand, it is worth pointing out that $\Psi_{\mathrm{RMSE},b}$ (6) is the most widely used error metric, even though it does not always accurately linearly quantify the error (e.g., Case 3 and Case 5 in Table 2). However, $\Psi_{\mathrm{RMSE},a}$ and $\Psi_{\mathrm{RMSE},b}$ (6) are the most straightforward extensions of the single-value estimation to a multiple-value estimation.

Let us now consider that the performance of a DoA estimator is always evaluated in the statistical sense with respect to different noise realizations, which are also approached as trials or observations. Therefore, Equations (4)–(6) are modified by taking into account *N* Monte-Carlo trials:

(7) $$\begin{array}{ccc} \Psi_{\mathrm{ME}} & \;\;=\;\; & \frac{1}{MN}\sum_{n=1}^{N}\sum_{m=1}^{M}\left({\hat{\psi }}_{m,n}-\psi_{m,n}\right)\\ \Psi_{\mathrm{MAE}} & \;\;=\;\; & \frac{1}{MN}\sum_{n=1}^{N}\sum_{m=1}^{M}\left|{\hat{\psi }}_{m,n}-\psi_{m,n}\right| \end{array}$$

(8) $$\begin{array}{ccc} \Psi_{\mathrm{MSE}} & \;\;=\;\; & \frac{1}{MN}\sum_{n=1}^{N}\sum_{m=1}^{M}{\left({\hat{\psi }}_{m,n}-\psi_{m,n}\right)}^{2} \end{array}$$

(9) $$\begin{array}{ccc} \Psi_{\mathrm{RMSE},c} & \;\;=\;\; & \frac{1}{MN}\sum_{n=1}^{N}\sum_{m=1}^{M}\sqrt{{\left({\hat{\psi }}_{m,n}-\psi_{m,n}\right)}^{2}}\\ \Psi_{\mathrm{RMSE},d} & \;\;=\;\; & \sqrt{\frac{1}{MN}\sum_{n=1}^{N}\sum_{m=1}^{M}{\left({\hat{\psi }}_{m,n}-\psi_{m,n}\right)}^{2}}\\ \Psi_{\mathrm{RMSE},e} & \;\;=\;\; & \frac{1}{M}\sum_{m=1}^{M}\sqrt{\frac{1}{N}\sum_{n=1}^{N}{\left({\hat{\psi }}_{m,n}-\psi_{m,n}\right)}^{2}}\\ \Psi_{\mathrm{RMSE},f} & \;\;=\;\; & \frac{1}{N}\sum_{n=1}^{N}\sqrt{\;\frac{1}{M}\sum_{m=1}^{M}{\left({\hat{\psi }}_{m,n}-\psi_{m,n}\right)}^{2}}. \end{array}$$

In the literature, the metric $\Psi_{\mathrm{ME}}$ (7) is referred to as the Mean Error (ME), $\Psi_{\mathrm{MAE}}$ (7) as the mean absolute error (MAE) (e.g., [8,29] for 2D cases), $\Psi_{\mathrm{MSE}}$ (8) as the mean square error (MSE) [2], and $\Psi_{\mathrm{RMSE},c}$–$\Psi_{\mathrm{RMSE},f}$ (9) as the RMSE. Also, in this case, it is worth noting there are various definitions of RMSE [10]. For example, the definition of $\Psi_{\mathrm{RMSE},c}$ is hardly ever found in the literature as the definitions of $\Psi_{\mathrm{MAE}}$ and $\Psi_{\mathrm{RMSE},c}$ are equivalent due to the relationship $\sqrt{{\left({\hat{\psi }}_{m,n}-\psi_{m,n}\right)}^{2}}=\left|{\hat{\psi }}_{m,n}-\psi_{m,n}\right|$. $\Psi_{\mathrm{RMSE},d}$ is adopted in [30,31,32,33]. The definition of $\Psi_{\mathrm{RMSE},e}$ is adopted in [34] (p. 5500213) and [35] (Equation (44)), whereas $\Psi_{\mathrm{RMSE},f}$ is in [36] (p. 3378) and [37] (p. 7).

Other variations not reported here include a metric similar to $\Psi_{\mathrm{RMSE},d}$ but that considers 2MN instead of MN (e.g., [38] (p. 214), [39] (Equation (34)), and [40] (p. 2804), where the addition of the term ‘2’ is due to the 2D DoA estimation scenario) and a metric similar to $\Psi_{\mathrm{RMSE},f}$, but that also considers the cases where the number of estimated angles is different from the number of true angles [41] (Equation (15)). Since $\Psi_{\mathrm{ME}}$ and $\Psi_{\mathrm{MSE}}$ yield incorrect estimations in some cases (as shown in Table 2), RMSEs have gained popularity as widely accepted error metrics. It is recommended that $\Psi_{\mathrm{RMSE},a}$ and $\Psi_{\mathrm{RMSE},c}$ are the best metrics for 1D DoA estimation when N=1 and N>1, respectively, as the results of these metrics largely coincide with the $\Psi_{\mathrm{AE}}$, as shown in Table 2.

## 3. Error Metrics for 2D DoA Estimation

In 2D DoA estimation, the direction of arrival is characterized by two angles. Some authors (e.g., those in [17,18,19,20]) have proposed defining the error metric separately for each each angular coordinate θ and ϕ (as defined in a spherical reference system) by applying the 1D RMSE metric in (9) ($\Psi_{\mathrm{RMSE},d}$) independently for θ and for ϕ. The θ error and ϕ error are then defined separately:

(10) $$\begin{array}{c} \Psi_{\mathrm{RMSE},\theta }=\sqrt{\frac{1}{MN}\sum_{n=1}^{N}\sum_{m=1}^{M}{\left({\hat{\theta }}_{m,n}-\theta_{m,n}\right)}^{2}}\\ \Psi_{\mathrm{RMSE},\phi }=\sqrt{\frac{1}{MN}\sum_{n=1}^{N}\sum_{m=1}^{M}{\left({\hat{\phi }}_{m,n}-\phi_{m,n}\right)}^{2}}. \end{array}$$

Such an approach may not offer a comprehensive view of the error in 2D DoA estimation since (10) does not provide a joint error. To address this issue, other authors have combined the azimuth and the elevation error (e.g., [8] (Equation (11))):

(11) $$\Psi_{\mathrm{MAE}}^{2D}=\frac{1}{MN}\sum_{n=1}^{N}\sum_{m=1}^{M}\left(\right|{\hat{\theta }}_{m,n}-\theta_{m,n}\left|+\right|{\hat{\phi }}_{m,n}-\phi_{m,n}\left|\right)$$

or (e.g., [8] (Equation (12)), [9,10,21,22,23,24,25,26,27]):

(12) $$\Psi_{\mathrm{RMSE}}^{2D}\;=\;\;\sqrt{\;\;\frac{1}{MN}\;\;\sum_{n=1}^{N}\sum_{m=1}^{M}\;\;\left[{\left({\hat{\theta }}_{m,n}\;\;-\;\theta_{m,n}\right)}^{2}\;\;+\;{\left({\hat{\phi }}_{m,n}\;\;-\;\phi_{m,n}\right)}^{2}\right]}.$$

**Figure 3:**
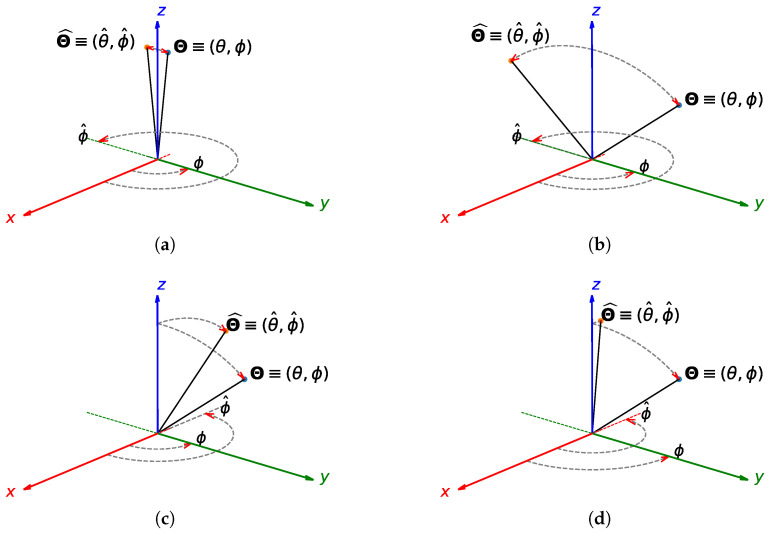
Representation of the test cases—(**a**) Case 1, (**b**) Case 2, (**c**) Case 3, and (**d**) Case 4.

The 2D superscript in this context indicates that the two angular errors are combined by treating them as a two-dimensional array index while ignoring the physical 3D problem. Both expressions, (11) and (12), sometimes provide unexpected results when, for instance, incoming signals arrive from directions close to the *z*-axis, as in Figure 3a. Indeed, even though the true and estimated angles are close (Figure 3a), the estimated error is surprisingly high (Table 3).

Furthermore, only a few papers (e.g., [7] (Equation (32)) and [42] (Equation (16))) consider a 3D error metric, which works as follows.

Let $\underline{\Theta \;}\;\equiv \left(\theta ,\phi \right)$ be the true DoA, while $\hat{\underline{\Theta \;}\;}\equiv \left(\hat{\theta },\hat{\phi }\right)$ is the estimated one (Figure 3). By expressing the angular directions in terms of cosine directions, we can define the unit vectors $\underline{u\;}\;$ and $\hat{\underline{u\;}\;}$:

$$\begin{array}{c} \underline{\Theta \;}\;\equiv \;\left(\theta ,\phi \right)↔\;\underline{u\;}\;\equiv \;\left(\sin\theta \cos\phi ,\sin\theta \sin\phi ,\cos\theta \right)\\ \hat{\underline{\Theta \;}\;}\equiv \left(\hat{\theta },\hat{\phi }\right)↔\;\hat{\underline{u\;}\;}\equiv \;\left(\sin\hat{\theta }\cos\hat{\phi },\sin\hat{\theta }\sin\hat{\phi },\cos\hat{\theta }\right). \end{array}$$

Then, the root square error in the cosine director space becomes

(13) $$\Psi_{\mathrm{RSE}}^{3D}=\sqrt{{\left({\hat{u}}_{x}-u_{x}\right)}^{2}+{\left({\hat{u}}_{y}-u_{y}\right)}^{2}+{\left({\hat{u}}_{z}-u_{z}\right)}^{2}}.$$

The 3D superscript underscores that we are considering a physical three-dimensional problem.

This error index (13) quantifies the Euclidean distance between two unit vectors, $\underline{u\;}\;$ and $\hat{\underline{u\;}\;}$, without direct information about the angular error. Therefore, the error in the angular domain can be defined (see [42] (Equation (16)) and [7] (Equation (32))):

(14) $$\begin{array}{c} \Psi_{\mathrm{AE}}^{3D}=\arccos\left(\underline{u\;}\;\cdot \hat{\underline{u\;}\;}\right)=\arccos\left(u_{x}{\hat{u}}_{x}+u_{y}{\hat{u}}_{y}+u_{z}{\hat{u}}_{z}\right) \end{array}.$$

As a matter of fact, (13) and (14) are related to each other, as

(15a) $$\begin{array}{ccc} \Psi_{\mathrm{AE}}^{3D} & = & \arccos\left(1-\frac{\Psi_{\mathrm{RSE}}^{3D^{2}}}{2}\right) \end{array}$$

(15b) $$\begin{array}{ccc} \mathrm{or},\;\;\Psi_{\mathrm{RSE}}^{3D} & = & \sqrt{\left(2-2\cos\Psi_{\mathrm{AE}}^{3D}\right)}. \end{array}$$

Although the units of (13) and (14) are different, it can be observed that they are approximately equivalent when the angular error is small; i.e., for $\Psi_{\mathrm{AE}}^{3D}<<1$, the cosine in (15b) can be expanded in series and (15b) can be simplified to $\Psi_{\mathrm{RSE}}^{3D}\approx \Psi_{\mathrm{AE}}^{3D}$, as in Appendix A. Finally, similarly to (11), the extension of $\Psi_{AE}^{3D}$ to *M* DoAs and *N* trials is given by

(16) $$\begin{array}{c} \Psi_{\mathrm{MAE}}^{3D}=\frac{1}{MN}\sum_{n=1}^{N}\sum_{m=1}^{M}\Psi_{{\mathrm{AE}}_{m,n}}^{3D}. \end{array}$$

For a comparative analysis of the error figures for 2D DoA estimation, let us consider the test cases shown in Figure 3. The results are summarized in Table 3. Clearly, the error defined in the cosine director’s space provides meaningful results in all cases.

An interesting point to note is that although [7] correctly identifies that minimizing the space angle (14) is the ultimate goal, the final proposed solution in [7] takes a different approach and maps the unit vectors $\underline{u\;}\;$ and $\hat{\underline{u\;}\;}$ differently, in a four component array [7] (Equation (36)), for the purpose of optimization. As a consequence, the error SE_2_ adopted in [7] (Equation (37)) fails miserably at quantifying the error for the cases shown in Figure 3. For instance, the metric SE_2_ [7] (Equation (37)) provides the same error value for the scenarios presented in Figure 3a,b, which is evidently a wrong and misleading result. In addition, SE_2_ provides a smaller error for the scenarios shown in Figure 3c,d compared to the one shown in Figure 3a, which is a completely wrong error estimation.

As a result, only [42] (Equation (16)) suggested an error metric which is equivalent to the metric we proposed in Equation (14). Therefore, the less commonly adopted error metric (14) and its extended version (16) are the correct ones and should be recommended for current and future 2D applications. In [42], the extended version of the metric (14) is not (16); instead, an RSME is calculated [42] (Equation (17)) which, using the notation of this paper, can be expressed as

(17) $$\Psi_{\mathrm{RMSE}}^{3D}=\sqrt{\frac{1}{MN}\sum_{n=1}^{N}\sum_{m=1}^{M}{\left(\Psi_{{\mathrm{AE}}_{m,n}}^{3D}\right)}^{2}}.$$

M=1 should be chosen since [42] (Equation (17)) considers only one direction of arrival. It is worth pointing out that Equations (16) and (17) are exactly equal (i.e., $\Psi_{\mathrm{MAE}}^{3D}=\Psi_{\mathrm{RMSE}}^{3D}$) when M=N=1. On the other hand, they provide different errors for any incorrect estimation when M>1 or N>1. For example, consider TC 2 to TC 4 in Table 3 and in Figure 3a–c, where there are three different estimations (i.e., N=3) of the same incoming DoA, $\underline{\Theta \;}\;\equiv \left(\theta ,\phi \right)=\left(45,90\right)$. In this case, the MAE and RSME error estimated with Equations (16) and (17) are $\Psi_{\mathrm{MAE}}^{3D}=65.06$ deg and $\Psi_{\mathrm{RMSE}}^{3D}=67.68$ deg, respectively, very close estimations, though not identical. Therefore, the proposed error metric $\Psi_{\mathrm{MAE}}^{3D}$ (16) is different from [42] (Equation (17)) and demonstrates all the necessary attributes for broad adoption in 2D DoA estimation. For completeness, Table 4 outlines the computational costs of the error metrics in terms of the number of operations performed. The proposed error metric requires approximately three times more operations than conventional metrics due to the additional computations of the unit vector components and their dot products.

## 4. Experimental Validation

In this section, the performance of the error metrics in different electromagnetic scenarios is analyzed with a locator [43] specifically designed for Bluetooth Low-Energy (BLE) applications. The details of the fabricated 3×3 array and its application for DoA estimation are reported in [43] for a single channel and in [44] for multiple channels of the BLE band. The DoAs are estimated with the classical MUltiple SIgnal Classification (MUSIC) method using the Conventional Steering Matrix (CSV) and Embedded Radiation Pattern (ERP), as discussed in [43].

### 4.1. Dependence of Incoming DoA

**Figure 4:**
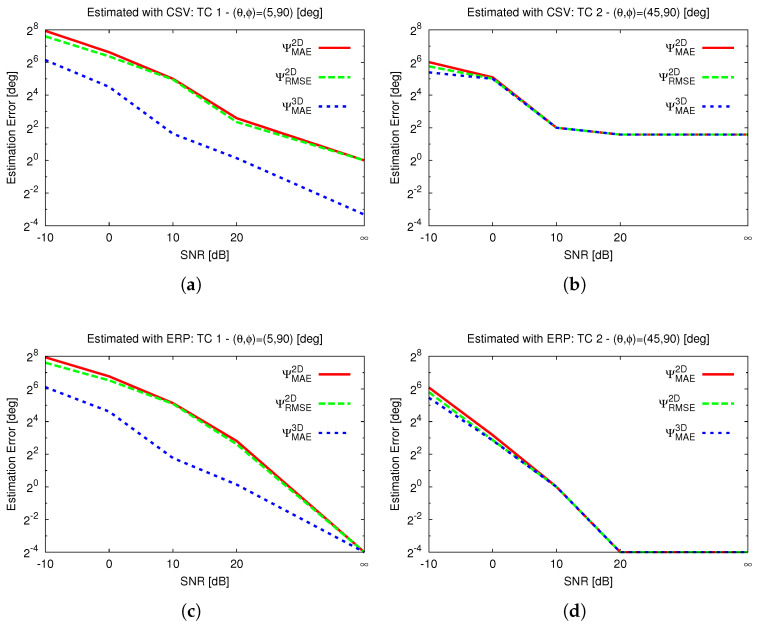
Error vs. SNR, estimated using different metrics: CSV method (**a**) TC 1 and (**b**) TC 2; ERP method (**c**) TC 1 and (**d**) TC 2.

In the first scenario analyzed, the sensitivity to the direction of the incoming signals, as indicated in TC 1 and TC 2 of Table 3 and Figure 3a,b, is analyzed within a real setup under varying noise levels, as shown in Figure 4.

Specifically, the estimation error for TC 1 when using the CSV approach is shown in Figure 4a and that when using the ERP is shown in Figure 4c, while the error for TC 2 is presented in Figure 4b when using the CSV approach and Figure 4d when using the ERP. As expected and confirmed by [43], the ERP outperforms the CSV in DoA estimation in terms of both metrics; however, the proposed metric ($\Psi_{\mathrm{MAE}}^{3D}$) consistently produces lower estimated errors compared to commonly used metrics across the different cases and in noisy scenarios. The errors evaluated using the 2D metrics are, in general, overestimated for the θ≈0 case compared to the one found with the proposed 3D metric, reinforcing the results in Table 3. Finally, the estimated errors for TC 2 are less sensitive to the choice of error metric, which aligns with the findings in Table 3 for some DoAs (i.e., TC 2 to TC 4). This suggests that 2D error metric definitions may exaggerate the error in DoAs from the broadside (θ≈0) direction.

### 4.2. Reference Frame Dependence

**Figure 5:**
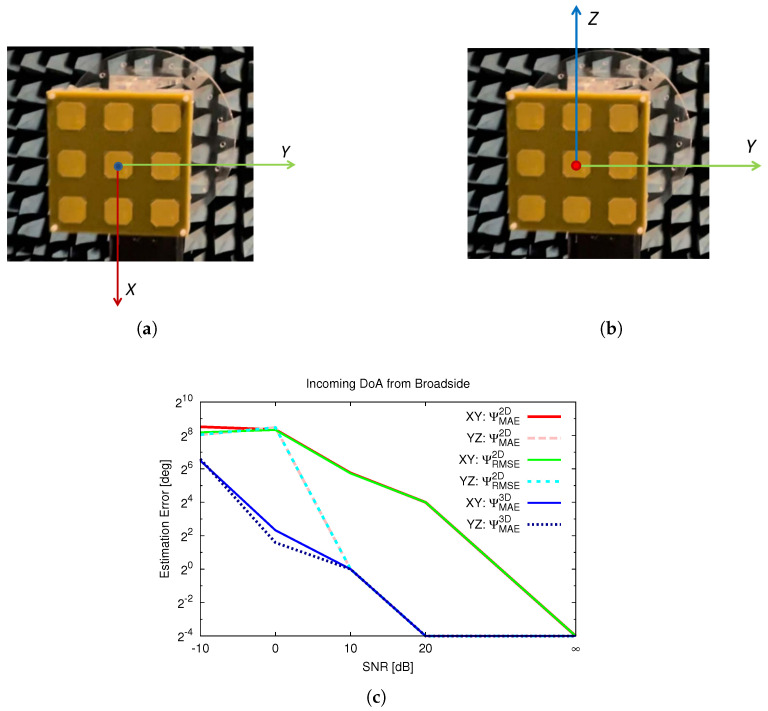
Frame dependence—(**a**) locator in XY plane, (**b**) locator in YZ plane, and (**c**) errors evaluated using different metrics.

To verify the universality of the metric across reference systems, an analysis was performed using the same physical direction of arrival but adopting different conventions for the reference system, specifically by using the antenna’s plane, labeled as the YZ plane, as illustrated in Figure 5b, instead of the previously considered XY plane, as shown in Figure 5a. Thus, the same incoming signal from the broadside direction will have the direction of arrival (θ,ϕ)=(0,0) as the first choice, Figure 5a, when the antenna array lays in the XY plane, and (θ,ϕ)=(90,0) as the second choice, Figure 5b, when the the antennas lay in the YZ plane. The estimated errors obtained from the different metrics at varying noise levels are presented in Figure 5c. Notably, the proposed metric, $\Psi_{\mathrm{MAE}}^{3D}$, demonstrates a consistent error across reference systems, while other metrics show sensitivity to the different reference systems, as highlighted in Figure 5c.

### 4.3. Dependence on Number of Trials

**Figure 6:**
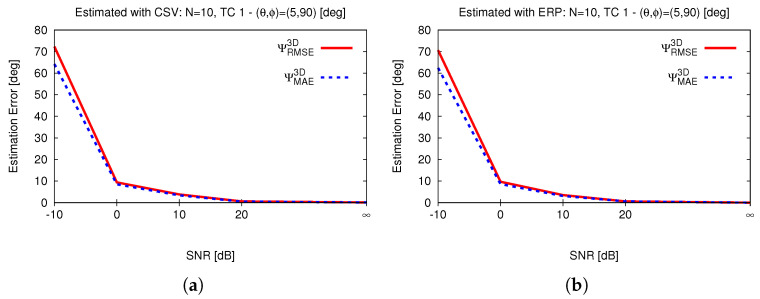
Sensitivity to number of trials—(**a**) CSV and (**b**) ERP.

The proposed metric, $\Psi_{\mathrm{MAE}}^{3D}$ (Equation (16)), is not exactly equal to $\Psi_{\mathrm{RMSE}}^{3D}$ (in Equation (17)), which is an extended version of Equation (14) reported in [42] (Equation (17)), at M=1, although $\Psi_{\mathrm{MAE}}^{3D}=\Psi_{\mathrm{RMSE}}^{3D}$ when M=N=1. Therefore, the number of DoAs (*M*) and the number of trials (*N*) both parameters that these error metrics are sensitive to when M≠1 or N≠1. Figure 6 shows the dependence of error estimation on the number of trials, *N*, with the proposed metric (Equation (16)) and with the metric reported in [42] (Equation (17)), which is equal to the metric $\Psi_{\mathrm{RMSE}}^{3D}$ (Equation (17)), when M=1. It is clearly evident that the error metric discussed in [42] (Equation (17)) and the newly proposed one are very similar but not exactly equal.

## 5. Potential Use Cases

The proper definition of an error metric is not only important for evaluating the performance of an estimator, but it also plays an important role in improving the accuracy of some iterative methods [45,46], learning-based methods [47,48,49], and hybrid methods [47].

Iterative algorithms first perform a preliminary estimation using approximations, reduced angular samples, or fewer snapshots in order to provide a quick estimation. The output of that preliminary estimation is then fed into an iterative loop in order to refine the result. To this end, some algorithms utilize directly estimated DoAs, some utilize estimation errors, some utilize both estimated DoAs and estimated errors, and, last but not least, some utilize other quantities taken from either the estimated DoAs or estimated error or both. Therefore, the choice of error metric is crucial, especially for 2D DoA estimation in iterative algorithms. The fundamental steps of an iterative estimator include the following:

1. Collect data (e.g., array signal snapshots).
2. Estimate quick/rough DoAs.
3. Compute DoA estimation error by means of a proper error metric for 2D DoAs.
4. Refine data (i.e., by noise cancellation) or angular range (i.e., by defining the range of the estimated DoA) or angular samples (i.e., by defining a dense grid for higher resolution) based on the estimated DoAs and estimated errors.
5. Restimate the DoA with the refined data or angular range or angular samples.
6. Continue 3–5 until the stopping criteria are reached.

It is understood that in this context, the error to be evaluated can be either between the estimated DoA and the ground truth or between two successive estimations (refinements).

In addition, the DoA estimation error serves as a critical component during the training phase of machine learning-based DoA estimators. It is used in multiple ways to improve model accuracy, robustness, and generalization. In supervised learning approaches, estimation errors can be used as ground truth labels to train deep learning models [48] such as convolutional neural networks (CNNs). During the training phase, a CNN algorithm learns to predict the DoA by minimizing the estimation error quantified using an error metric (i.e., loss function). By optimizing this loss function, the network adjusts its parameters to reduce the estimation error, thereby improving its predictive performance. Moreover, in reinforcement learning (RL), DoA estimation error serves as a reward. The model iteratively refines DoA predictions by minimizing the estimation error and maximizing the reward. For example, Deep Q-Networks (DQNs) [49] are used to adjust beamforming parameters based on estimated errors.

The estimation error obtained by means of a proper error metric is a key component of hybrid DoA estimation methods [47], which combine multiple techniques, such as classical and machine learning-based approaches. The critical steps of a hybrid estimator include the following:

1. Collect training data (e.g., array signal snapshots) on known true DoAs.
2. Estimate DoAs using conventional methods (e.g., MUSIC).
3. Compute the DoA estimation error by means of a proper error metric for 2D DoAs.
4. Train a leaning method (e.g., a CNN) to predict DoA correction factors based on input features.
5. Apply the correction to improve DoA estimation accuracy.

From the above discussion, it is clear that the choice of error metric is a critical one that influences various aspects of iterative methods and machine learning, including stopping criteria, reward evaluations, and dataset training.

## 6. Conclusions

The error metrics used in the SoA literature on DoA estimation have been reviewed thoroughly and a “proper and unique” error metric for 2D DoA estimation has been recommended. In recent papers, a proper definition of the error metric used is often omitted, and, in some cases, inappropriate ones that lack a meaningful physical interpretation are adopted. It has been observed that commonly used error metrics can sometimes yield incorrect and misleading results due to the physical 3D nature of the 2D DoA estimation problem. Therefore, we proposed an error-free universal error metric, which was defined by exploiting the cosine directors in Equation (14). In addition, it has been verified, using numerous representative examples, that the proposed new error metric is able to provide meaningful results as it is related to the angular difference between the actual direction of arrival and its estimation. Finally, the sensitivity of different error metrics were validated using the experimental data from a fabricated 3×3 locator designed for BLE applications. The experimental validation confirmed that the proposed metric is insensitive to the direction of incoming signals, the reference system used, and the number of trials conducted compared to other well-known error metrics.

## Figures and Tables

**Table 1:** Numerical analysis of error seen in simple DoA estimation cases.

TC	ψ	$\hat{\psi }$	$\Psi_{E}$	$\Psi_{\mathrm{SE}}$	$\Psi_{\mathrm{RSE}}$
1	10	5	−5	25	5
2	10	10	0	0	0
3	10	15	5	25	5

**Table 2:** Numerical analysis of error for M=3 values.

TC	ψ	$\hat{\psi }$	$\Psi_{\mathrm{AE}}$	$\Psi_{\mathrm{ASE}}$	$\;\;\Psi_{\mathrm{RMSE},a}\;\;$	$\;\;\Psi_{\mathrm{RMSE},b}\;\;$
1	[5, 10, 15]	[4, 9, 14]	−1	1	1	1
2	[5, 10, 15]	[5, 10, 15]	0	0	0	0
3	[5, 10, 15]	[5, 9, 16]	0	0.67	0.67	0.82
4	[5, 10, 15]	[10, 15, 20]	5	25	5	5
5	[5, 10, 15]	[20, 10, 15]	5	75	5	8.66

**Table 3:** Numerical analysis of error in 2D DoA estimation.

TC	$\underline{\Theta \;}\;\equiv \left(\theta ,\phi \right)$	$\hat{\underline{\Theta \;}\;}\equiv \left(\hat{\theta },\hat{\phi }\right)$	$\Psi_{\mathrm{MAE}}^{2D}$	$\Psi_{\mathrm{RMSE}}^{2D}$	$\Psi_{\mathrm{MAE}}^{3D}$
1	(5, 90)	(5, 270)	180	180	10
2	(45, 90)	(45, 270)	180	180	90
3	(45, 90)	(45, 180)	90	90	60
4	(45, 90)	(5, 180)	130	98.5	45.2

**Table 4:** Computational complexity in terms of number of operations.

Error Metric	Complexity	Example: M=N=1
$\Psi_{\mathrm{MAE}}^{2D}$	O(5MN+(MN−1)+1)	6
$\Psi_{\mathrm{RMSE}}^{2D}$	O(5MN+(MN−1)+2)	7
$\Psi_{\mathrm{MAE}}^{3D}$	O(18MN+(MN−1)+1)	19

## Data Availability

The original contributions presented in this study are included in the article.

## Abbreviations

The following abbreviations are used in this manuscript:

DoA	Direction of Arrival
RMSE	Root Mean Square Error
MSE	Mean Square Error
MAE	Mean Absolute Error
IoT	Internet of Things
UAV	Unmanned Aerial Vehicle
IEEE	Institute of Electrical and Electronics Engineers
5G	Fifth Generation
6G	Sixth Generation
CRB	Cramér–Rao Bound
CDF	Cumulative Distributed Function
TC	Test Case
1D	One-Dimensional
2D	Two-Dimensional

## Appendix A

This appendix presents the derivation of the key relationships between the RSE and AE metrics.

Starting from the definition of the RSE error, (13), we express the squared RSE error, ${\left(\Psi_{\mathrm{RSE}}^{3D}\right)}^{2}$, in terms of the dot product of the unit vectors $\underline{u\;}\;$ and $\hat{\underline{u\;}\;}$:

(A1) $$\begin{array}{c} {\left(\Psi_{\mathrm{RSE}}^{3D}\right)}^{2}={\left({\hat{u}}_{x}-u_{x}\right)}^{2}+{\left({\hat{u}}_{y}-u_{y}\right)}^{2}+{\left({\hat{u}}_{z}-u_{z}\right)}^{2}={\left|\underline{u\;}\;-\hat{\underline{u\;}\;}\right|}^{2}=\\ \;\;\;\;\;\;\;=\left(\underline{u\;}\;-\hat{\underline{u\;}\;}\right)\cdot \left(\underline{u\;}\;-\hat{\underline{u\;}\;}\right)=\underline{u\;}\;\cdot \underline{u\;}\;-\hat{\underline{u\;}\;}\cdot \underline{u\;}\;-\underline{u\;}\;\cdot \hat{\underline{u\;}\;}+\hat{\underline{u\;}\;}\cdot \hat{\underline{u\;}\;}=2-2\underline{u\;}\;\cdot \hat{\underline{u\;}\;} \end{array}$$

Similarly, from the definition of the AE error, (14), we obtain a direct expression of the cosine of the angular error

(A2) $$\cos\left(\Psi_{\mathrm{AE}}^{3D}\right)=u_{x}{\hat{u}}_{x}+u_{y}{\hat{u}}_{y}+u_{z}{\hat{u}}_{z}=\underline{u\;}\;\cdot \hat{\underline{u\;}\;}$$

To derive the relationship, (15), linking the RSE and AE errors, we combine (A1) and (A2)

(A3) $${\left(\Psi_{\mathrm{RSE}}^{3D}\right)}^{2}=2-2\cos\left(\Psi_{\mathrm{AE}}^{3D}\right)$$

$$\frac{{\left(\Psi_{\mathrm{RSE}}^{3D}\right)}^{2}}{2}=1-\cos\Psi_{\mathrm{AE}}^{3D}$$

(A4) $$\cos\Psi_{\mathrm{AE}}^{3D}=1-\frac{{\left(\Psi_{\mathrm{RSE}}^{3D}\right)}^{2}}{2}$$

by inverting the last expression (A4), we obtain (15a):

$$\Psi_{\mathrm{AE}}^{3D}=\arccos\left(1-\frac{\Psi_{\mathrm{RSE}}^{3D^{2}}}{2}\right)$$

by taking the square root of (A3) we obtain (15b):

$$\Psi_{\mathrm{RSE}}^{3D}=\sqrt{\left(2-2\cos\Psi_{\mathrm{AE}}^{3D}\right)}.$$
